# The Natural Product Domain Seeker NaPDoS: A Phylogeny Based Bioinformatic Tool to Classify Secondary Metabolite Gene Diversity

**DOI:** 10.1371/journal.pone.0034064

**Published:** 2012-03-29

**Authors:** Nadine Ziemert, Sheila Podell, Kevin Penn, Jonathan H. Badger, Eric Allen, Paul R. Jensen

**Affiliations:** 1 Center for Marine Biotechnology and Biomedicine, Scripps Institution of Oceanography, University of California San Diego, La Jolla, California, United States of America; 2 Microbial and Environmental Genomics, J. Craig Venter Institute, San Diego, California, United States of America; 3 Marine Biology Research Division, Scripps Institution of Oceanography, University of California San Diego, La Jolla, California, United States of America; University of Florida, United States of America

## Abstract

New bioinformatic tools are needed to analyze the growing volume of DNA sequence data. This is especially true in the case of secondary metabolite biosynthesis, where the highly repetitive nature of the associated genes creates major challenges for accurate sequence assembly and analysis. Here we introduce the web tool Natural Product Domain Seeker (NaPDoS), which provides an automated method to assess the secondary metabolite biosynthetic gene diversity and novelty of strains or environments. NaPDoS analyses are based on the phylogenetic relationships of sequence tags derived from polyketide synthase (PKS) and non-ribosomal peptide synthetase (NRPS) genes, respectively. The sequence tags correspond to PKS-derived ketosynthase domains and NRPS-derived condensation domains and are compared to an internal database of experimentally characterized biosynthetic genes. NaPDoS provides a rapid mechanism to extract and classify ketosynthase and condensation domains from PCR products, genomes, and metagenomic datasets. Close database matches provide a mechanism to infer the generalized structures of secondary metabolites while new phylogenetic lineages provide targets for the discovery of new enzyme architectures or mechanisms of secondary metabolite assembly. Here we outline the main features of NaPDoS and test it on four draft genome sequences and two metagenomic datasets. The results provide a rapid method to assess secondary metabolite biosynthetic gene diversity and richness in organisms or environments and a mechanism to identify genes that may be associated with uncharacterized biochemistry.

## Introduction

Genome sequencing has revealed that the secondary metabolite potential of even well studied bacteria has been severely underestimated [1], [2]. This revelation has led to an explosion of interest in genome mining as an approach to natural product discovery [3], [4], [5], [6], [7], [8]. Considering that natural products remain one of the primary sources of therapeutic agents [9], [10], sequence analysis provides opportunities to identify strains with the greatest genetic potential to yield novel secondary metabolites prior to chemical analysis and thus increase the rate and efficiency with which new drug leads are discovered. In addition, community or metagenomic analyses can be used to identify environments with the greatest secondary metabolite potential and to address ecological questions related to secondary metabolism. To capitalize on these opportunities, it is critical that new bioinformatics tools be developed to handle the massive influx of sequence data that is being generated from next generation sequencing technologies [11].

Polyketide synthases (PKSs) and non-ribosomal peptide synthetases (NRPSs) are large enzyme families that account for many clinically important pharmaceutical agents. These enzymes employ complimentary strategies to sequentially construct a diverse array of natural products from relatively simple carboxylic acid and amino acid building blocks using an assembly line process [12], [13]. The molecular architectures of PKS and NRPS genes have been reviewed in detail and minimally consist of activation (AT or A), thiolation (ACP or PCP), and condensation (KS or C) domains, respectively [14], [15], [16], [17], [18]. These genes are among the largest found in microbial genomes and can include highly repetitive modules that create considerable challenges to accurate assembly and subsequent bioinformatic analysis [8].

When the challenges associated with PKS and NRPS gene assembly can be overcome, a number of effective bioinformatics tools have been developed for domain parsing [19], [20] and domain string analysis [21], [22]. In cases of modular type I PKSs and NRPSs where domain strings follow the “co-linearity rule” such that substrates are incorporated and processed according to the precise domain organization observed in the pathway, bioinformatics has been used to make accurate structural predictions about the metabolic products of those pathways [23]. However, the increasing number of exceptions to co-linearity, such as module skipping and stuttering [24], create limitations for precise, sequence-based structure prediction. The bioinformatic tools currently available for secondary metabolism have been reviewed [25], [26] and are complemented by the recent release of antiSMASH, which has the capacity to accurately identify and provide detailed sequence analysis of gene clusters associated with all known secondary metabolite chemical classes [27]. While all of these tools have useful applications, NaPDoS employs a phylogeny based classification system that can be used to quantify and distinguish KS and C domain types from a variety of datasets including the incomplete genome assemblies typically obtained using next generation sequencing technologies. These specific domains were selected because they are highly conserved and have proven to be among the most informative in a phylogenetic context [28], [29].

Phylogenomics provides a useful approach to infer gene function based on phylogenetic relationships as opposed to sequence similarities [30], [31]. While the evolutionary histories of PKS and NRPS genes are largely uninformative due to their size and complexity, KS and C domain phylogenies reveal highly supported clustering patterns. These patterns have been used to distinguish type II PKSs associated with spore pigment and antibiotic biosynthesis [32], type I modular and hybrid PKSs [33], and subsequently to identify many different PKSs types [34]. KS phylogeny has also been used to predict pathway associations [26], [35] and, in some cases, the secondary metabolic products of those pathways [28], [36], [37]. Phylogenetics has also been used to successfully identify PKS sequences from complex metagenomic datasets [38]. Likewise, C domain phylogeny clearly delineates functional subtypes as opposed to species relationships [39] and has been used to identify new functional classes, such as the “starter” C domain [29]. Taken together, the established phylogenetic relationships of KS and C domains provide an effective framework within which to assess secondary metabolite gene richness and diversity and to identify new functional classes that may be associated with uncharacterized biosynthetic mechanisms.

Here we introduce the web tool Natural Product Domain Seeker (NaPDoS), which extracts and rapidly classifies KS and C domains from a wide range of sequence data. The results can be used to assess the potential for PKS and NRPS secondary metabolite biosynthesis in organisms or environments and to identify new phylogenetic lineages, which can subsequently be investigated as a source of new mechanistic biochemistry. We tested NaPDoS on four draft bacterial genome sequences and two metagenomic datasets. The results reveal a remarkable level of secondary metabolite gene diversity among closely related strains and provide a mechanism to assess secondary metabolism from poorly assembled genomic data.

## Materials and Methods

### Reference database

KS and C domains were extracted from select PKS and NRPS genes associated with experimentally characterized biosynthetic pathways using the online program NRPS-PKS (http://www.nii.res.in/searchall.html) [19], [21]. The pathways selected include representatives of the currently known enzyme architectures and functions associated with type I and II PKSs and NRPSs and thus this database is not meant to be comprehensive. The biochemical function and enzyme architecture of each domain was manually confirmed by analysis of the associated domain string and secondary metabolic product. Based on these results, each sequence was preliminarily assigned to a domain class. The compound produced by the associated pathway, the literature reference including PubMed ID, and the gene accession number was also recorded for each domain.

### Sequence alignment and phylogeny

The amino acid sequences of all reference KS and C domains were aligned using either MUSCLE [40] or ClustalX (version 1.83) [41] with the BLOSUM 62 protein weight matrix. The alignments were manually adjusted using Mesquite [42]. Maximum likelihood, parsimony, and neighbor-joining phylogenetic trees were constructed using the “a la carte” mode at the Phylogeny.fr website (http://www.phylogeny.fr/) [43]. Final maximum likelihood trees were constructed from the reference data set with the program PHYML [44]. Final domain classifications were made based on the phylogenetic relationships observed in these trees.

### NaPDoS and Webportal

**Figure 1:**
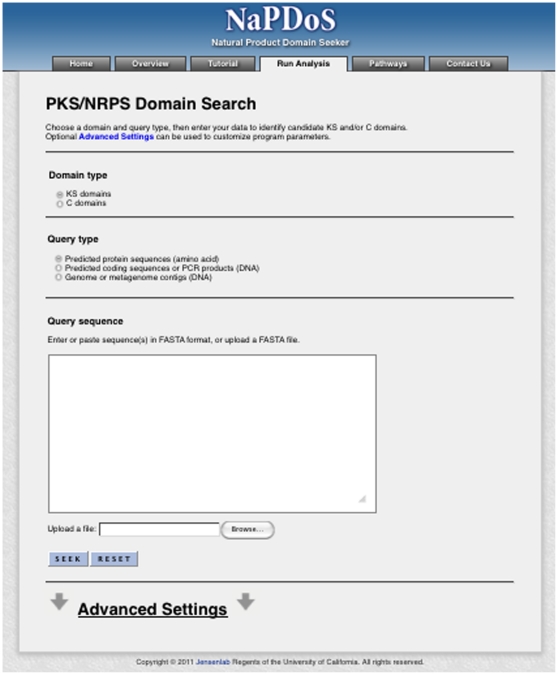
NaPDoS bioinformatic pipeline. The web interface to this pipeline is divided 3 consecutive steps. Nucleic acid sequences are translated into predicted amino acids and genomic sequences are screened using Hidden Markov Models (HMM). For protein and small nucleic acid sequences a BLAST search is performed against curated reference database examples to identify matches to known PKS/NRPS pathways. Selected candidate sequences plus the BLAST results are trimmed and inserted into a manually curated reference alignment, keeping the original reference alignment intact. This alignment is used to build a tree.

The NaPDoS web portal identifies candidate KS and C domains through a combination of hidden markov model (HMM) searches and the basic local alignment search tool (BLAST) algorithm [45] optimized for query input type as shown in Figure 1. PCR products or coding sequences (CDS) in nucleotide or amino acid format are analyzed directly by local BLASTX or BLASTP searches against the manually curated reference database of experimentally verified KS and C domains described above. This BLAST-based approach proved more effective than HMM models in detecting the target domains from short query sequences. Genomic sequences (including contigs, incomplete drafts, or complete genomes) and metagenomic nucleotide data sets are first pre-screened to obtain rough coordinates for KS and C domains using the KS domain HMM developed by Yadav and co-workers [21] and the PFAM C domain model PF00668 [46]. The resulting candidate domains are then subjected to BLAST analyses using the same manually curated reference database as described above.

BLAST results are linked to a back-end MySQL relational database via CGI-scripting to retrieve and report domain classification and related pathway information. Query sequences are trimmed according to their BLAST match coordinates by a custom Perl script then aligned to each other and their database matches using MUSCLE [40]. Trimmed sequences can be downloaded along with best BLAST matches in FASTA or MSF aligned format. Finally, trimmed and aligned candidate KS and C sequences plus BLAST matches can be inserted into a phylogenetic tree generated from the reference database using FastTree to estimate maximum likelihood [44]. Newick format output from FastTree is converted to SVG format graphic images using the Newick-Utilities program [47]. NaPDoS does not employ any stand-alone software that was created specifically for its operation but instead employs pre-existing and publically available programs as described above.

### Draft genomes and metagenomes

Draft genome sequences of *S. arenicola* strain CNH-643 (accession number PRJNA84391), *S. arenicola* strain CNT-088 (accession number PRJNA84269), *“S. pacifica”* strain CNS-143 (accession number PRJNA84389), and *“S. pacifica”* strain CNT-133 (accession number PRJNA84271) were obtained at 8× coverage at the J. Craig Venter Institute using 454 GS FLX pyrosequencing and 0.5× Sanger sequencing as previously described [48] based on an estimated genome size of 5.6 Mb. The sequence data were assembled using the Newbler Assembler with the mapping option [49]. *S. arenicola* strains were mapped onto the complete *S. arenicola* strain CNS-205 genome and the *S. pacifica* strains were mapped to the complete *S. tropica* CNB-440 genome [50] while any unmapped sequence data was assembled de novo. The four draft *Salinispora* genomes were mined for KS and C domains using NaPDoS with default settings. The metagenomic datasets (whale fall, AAFZ00000000, AAFY00000000, AAGA00000000 and Minnesota farm soil, AAFX00000000, [51]) were mined using default HMM settings (e^−5^) and the resulting sequences further subjected to a loose BLAST analysis with an e-value cut-off of 1 to obtain more precise coordinates and assign initial domain classifications.

## Results

### The Natural Product Domain Seeker (NaPDoS)

**Figure 2:**
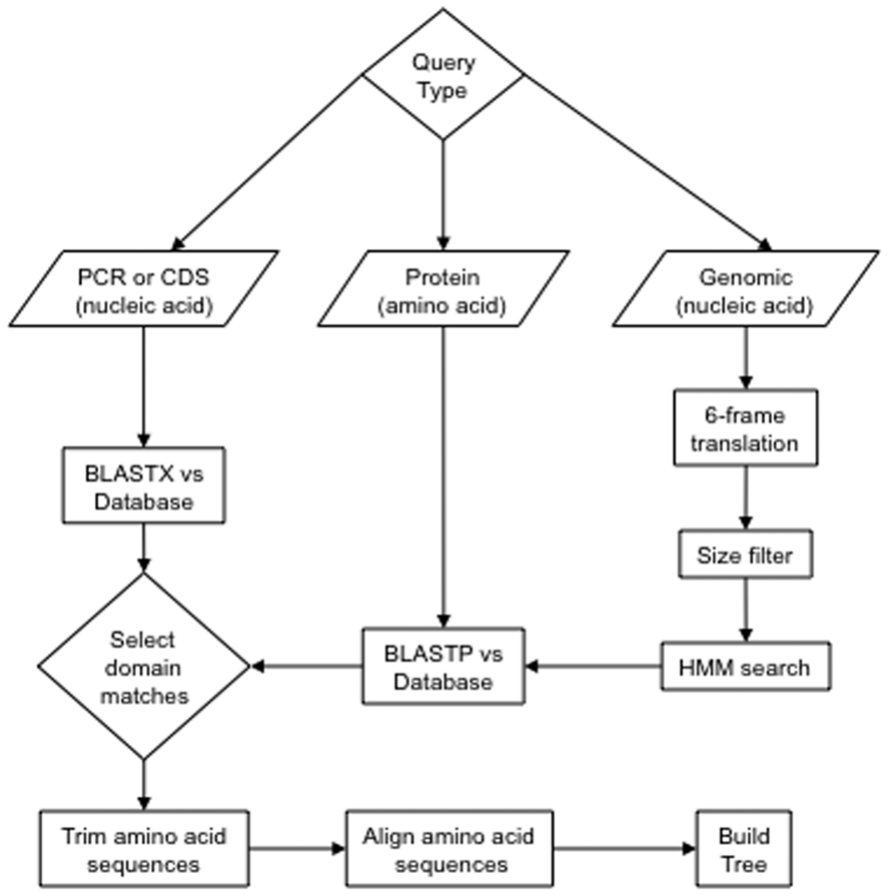
Screen shot of the NaPDoS webpage.

The publically available web tool NaPDoS (http://npdomainseeker.ucsd.edu/) was created to detect and classify KS and C domains in nucleotide and amino acid sequence data. The query data can be PCR amplicons, genes, contigs, genomes, or metagenomes. The current query size limits are <30 MB and <50,000 individual sequences. The website provides a detailed tutorial on the use of this tool, which is implemented using a web interface (Figure 2) that follows the bioinformatic pipeline shown in Figure 1. Query sequences are BLASTed against the reference database, which currently contains 459 KS and 190 C domains derived from 66 PKS, 20 NRPS, 8 PKS/NRPS hybrid, and 5 fatty-acid synthase (FAS) biosynthetic pathways. These sequences can be downloaded from the website and encompass all major classes of type I and II KS and C domains currently described in the literature [12], [29], [52], [53]. This manually curated database will be updated periodically as new modular architectures and biochemical features are discovered for each domain type.

The primary output for all analyses includes the query identification, best database match, percent identity, alignment length, e-value, and product and classification of the biosynthetic pathway associated with the best match. KS and C domain sequences derived from the input data can then be output in raw format or aligned with the best BLAST matches. A NaPDoS independent BLAST of the output domain sequence(s) against the NCBI nr database is also highly recommended to check for matches that do not occur in the reference database.

To generate a final classification for each domain sequence, it is highly recommended to construct a phylogenetic tree, especially in cases where the percent sequence identity to the top database match is low. If that option is chosen, a profile alignment is generated in which the query sequences are incorporated into a carefully curated reference alignment generated from the sequences in the reference database. This alignment is then used to create a phylogenetic tree, which needs to be manually interpreted to establish a final classification for each sequence. Interpreting sequences in the context of a phylogenetic tree is particularly important given that the NaPDoS pipeline is intentionally set to low stringency in an effort to detect all possible KS and C domains. Thus, homologs not involved in secondary metabolism such KSs associated with fatty acid biosynthesis are regularly detected. These sequences can be readily classified based on the phylogenetic tree.

### Domain classification

**Figure 3:**
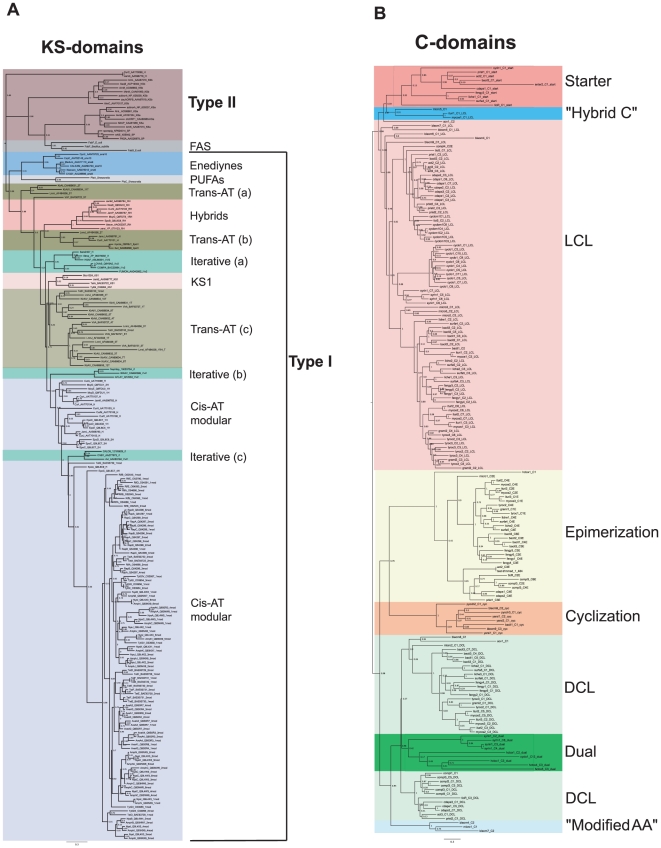
Phylogeny based domain classification. A) KS domain phylogeny. Polyphyletic groups are distinguished by letters. B) C domain phylogeny.

KS and C domain phylogenies form the basis of the NaPDoS classification system (Figure 3). KS domains clade based on biochemical function and enzyme architecture, which are described in Table 1. In some cases, e.g. enediynes, these clades are also predictive of structural motifs associated with the secondary metabolites produced. The KS phylogeny clearly delineates type I and II PKSs (Figure 3A). The shared ancestry reported between type II PKS and FAS sequences [54] is clearly maintained in this tree. The vast majority of the reference sequences fall into the type I PKS clade. This clade can be further resolved into seven classes, which are not always monophyletic. This polyphyly reflects the complex evolutionary histories of the different classes such as the *trans*-AT KSs, which evolved by extensive HGT and exploit considerably greater modular architectures than the *cis*-AT group, which has largely evolved by gene duplication [55]. However, all of these lineages are highly supported in the tree (likelihood values 0.7–1.0) and largely agree with previous phylogenetic studies [29], [56].

**Table 1:** KS domain classification.

Type	Class	Description	Product (example)
I	Enediyne	Iteratively acting, builds typical 9 or 10 membered enedyines.	Enediyne (calicheamicin)
	*Trans*-AT	Module lacks cognate AT domain; this activity is provided instead by a discrete protein encoded in *trans*.	Polyketide/macrolide(leinamycin)
	*Cis*-AT	Multi-domain module that includes AT domain.	Polyketide/macrolide (erythromycin)
	Hybrid	Catalyzes a condensation reaction between an amino acid and an acyl extender unit in a NRPS/PKS pathway.	Peptide-polyketide(microcystin)
	Iterative	Domain is used multiple times in a cyclic fashion.	Polycyclic polyketide(aflatoxin)
	PUFA	Produces long chain fatty acids containing more than one double bond.	Polyunsaturated fatty acid(omega-3-fatty-acid)
	KS1	Occurs in the first module of multimodular genes, includes typical starter KSs (KSQ) as well as KS domains that incorporate unusual precursors.	Polyketide, peptide-polyketide(salinosporamide)
II	Type II	Each domain occurs on a discrete protein.	Aromatic polyketide (actinorhodin)
	FAS	Involved in fatty acid biosynthesis (eg., FabB and FabF from bacteria).	Fatty acid(palmitic acid)

In the case of C domains, the sequences generally clade based on substrate specificity, i.e. the stereochemistry of the amino acids incorporated and the subsequent tailoring reactions they perform (Figure 3B). Eight clades are identified in the tree of which six are functionally characterized. The characterized clades are comprised of LCL domains, which catalyze a peptide bond between two L-amino acids, DCL domains, which link an L-amino acid to a growing peptide ending with a D-amino acid, starter C domains, which acylate the first amino acid with a β-hydroxy-carboxylic acid, cyclization domains, which catalyze both peptide bond formation and the subsequent cyclization of cysteine, serine or threonine residues, epimerization (E) domains, which switch the chirality of the last amino acid in the growing peptide, and dual E/C domains, which catalyze both epimerization and condensation reactions. These six functional classes are well supported in the tree and largely monophyletic. Two experimentally uncharacterized clades are identified in the tree, one of which has been conditionally assigned the name “modified AA” (Figure 3B). This clade contains domains from the bleomycin and microcystin pathways. Although the biochemical function of these domains has not been experimentally defined, they appear to be involved in the modification of the incorporated amino acid, for example the dehydration of serine to dehydroalanine [57], [58]. C domains in the second functionally uncharacterized clade have been conditionally assigned the name “hybrid C”. The three sequences in this clade (micro5, ituri1, and mycos1) are each located downstream of an aminotransferase domain and appear to be involved in the condensation of an amino acid to an aminated polyketide resulting in a hybrid PKS/NRPS secondary metabolite. The phylogenetic relationships of the KS and C domains in the reference dataset form the basis of the NaPDoS classification system and provide a framework within which new clades and biochemical functions can be discovered.

### Genome analyses

As a positive control, NaPDoS was used to analyze the genome sequence of *Streptomyces avermitilis* strain MA-4680. This analysis revealed 67 KS and 15 C domains (Table S1), which encompass all of the PKS, NRPS, and hybrid PKS/NRPS gene clusters that were reported to contain these domains [59]. NaPDoS also correctly identified all of the KS and C domains in the complete genome sequences of *S. tropica* (strain CNB-440) and *S. arenicola* (strain CNS-205) [50]. NaPDoS was then tested on four draft *Salinispora* genome sequences. These low coverage drafts were generated using pre-Titanium 454 technology (avreage read length 244 bp) and yielded poor assemblies and a large number of contigs (Table S2). There was no evidence that any biosynthetic gene clusters had been completely assembled based on the analysis of flanking regions and comparisons with pathways that appeared common with the CNB-440 and CNS-205 sequences [50]. None-the-less, NaPDoS successfully detected 18–36 KS domains and 5–14 C domains in the un-annotated FASTA files generated for each of the four draft genomes (Table S2). More than half (56%) of these sequences showed no significant BLAST matches to domains associated with biochemically characterized biosynthetic genes and thus could not be linked to specific secondary metabolic products. More significantly, 8 KS and 9 C domains detected in the four draft sequences were not observed in the two closed *Salinispora* genomes (Table S3). These sequences (KS7-14 and C5-13) cover a broad range of domain classes and indicate considerable new biosynthetic potential among a group of closely related strains. Two C domains fell into the “Modified AA” clade, which has yet to be experimentally characterized. Given that the upstream A domain specifies serine in both cases, it can be predicted that this domain results in the incorporation of dehydrated serine (ie., dehyroalanine) into the non-ribosomal peptide. This hypothesis has not yet been tested, but is supported by the reference sequences in this clade, which perform similar dehydration reactions.

Interestingly, two KS domains with close matches (89% and 94%) to those associated with the biosynthesis of salinosporamide A [60] were observed in “*S. pacifica*” strain CNT-133. This was unexpected given that compounds in this series had previously been reported exclusively from *S. tropica* [61]. This observation subsequently led to the discovery of a new compound in the salinosporamide series [5] and a rare window into pathway evolution in two closely related bacterial species [37]. Furthermore, a KS domain that shares close sequence identity with domains involved in the biosynthesis of tylactone in *Streptomyces fradiae* [62] was detected in strain CNH-643 (Table S3). Subsequent chemical studies revealed the production of several new tylactone derivatives by this strain (unpublished data). The same four draft genome sequences were also analyzed using antiSMASH [27], a sophisticated pipeline that can make structure predictions for a diverse range of secondary metabolic pathways. While antiSMASH worked well on the two complete *Salinispora* genomes, NaPDoS consistently detected more KS domains in the draft genomes (Table S4). While this is not surprising given that NaPDoS is specifically designed for this purpose, it nonetheless highlights the value of the sequence tag approach when working with draft genome sequences that contain many unassembled contigs.

### Metagenomic analyses

NaPDoS was further tested on metagenomic data sets generated from a Minnesota farm soil and whale fall [51]. While the numbers of KS domains detected in both datasets are similar (Table S5), removing redundant sequences reveals a higher diversity of KS domains in the soil sample. The majority of the whale fall KS domains were classified as FASs suggesting they are associated with fatty acid biosynthesis. In contrast, nearly half of the KS domains detected in the Minnesota farm soil appear to be involved in secondary metabolite biosynthesis. These results are in agreement with a previous study in which these datasets were manually screened for type I PKSs [38]. All of the sequences shared <70% identity to the reference database or NCBI BLAST matches associated with experimentally characterized pathways and thus no predictions could be made about the structures of the potential secondary metabolic products. None-the-less, the majority of the KS domains detected could be rapidly classified by NaPDoS. The incorporation of these domains into a phylogenetic tree containing the reference sequences led to the reclassification of some and the prediction that others are functionally distinct from KS domains (Tables S6 and S7). These sequences were likely detected due to the low stringency at which the NaPDoS BLAST analyses were performed on the meta-data and is a positive indication that the KS analysis was comprehensive. The reclassification of some sequences emphasizes the importance of incorporating phylogeny into the analyses.

## Discussion

Rapid advances in DNA sequencing technologies are providing unprecedented opportunities to incorporate DNA sequence data into the natural product discovery process. The effective use of this information requires bioinformatic tools that can rapidly analyze large datasets in the context of a wide array of complex biosynthetic paradigms. While a number of excellent bioinformatic tools targeting secondary metabolism have been developed [22], [25], [27], they are largely predicated on accurate gene and operon assembly, something that has often proven challenging to obtain given the modular and highly repetitive nature of many genes involved in secondary metabolism [8]. This challenge can become especially problematic in the case of metagenomic analyses of complex microbial communities.

The Natural Product Domain Seeker (NaPDoS) is a web-based bioinformatic tool that was developed to detect and classify KS and C domains from a wide variety of sequence data. The use of domain sequence tags as proxies for the biosynthetic genes in which they reside is based on the well established and highly informative phylogentic relationships they maintain. These relationships form the foundation of the NaPDoS classification system and provide a rapid mechanism to delineate secondary metabolite biosynthetic gene richness and diversity within a genome or environmental sample. Short sequence tags (e.g. 600 bp) can be effectively analyzed using NAPDoS and thus minimum coverage, next generation sequence assemblies are well suited for this tool. The resulting estimates of biosynthetic potential can be used to guide more extensive sequencing efforts or targeted operon assembly. In cases where query sequences closely match domains derived from experimentally characterized biosynthetic pathways (eg., >90% sequence identity), it has proven possible to make accurate predictions about the structural class of the secondary metabolite(s) produced [37], [63]. The low stringency of the HMM searches and the ability to adjust the internal BLAST parameters provides opportunities to detect more highly divergent KS and C domains associated with secondary metabolism as well as domains that are not associated with secondary metabolism (e.g. fatty acid biosynthesis) and thus all results should be carefully scrutinized. As the number of experimentally characterized biosynthetic pathways increases, this approach will provide an increasingly effective method to “de-replicate”, i.e. to identify strains that have the greatest potential to produce known compounds.

There is ample evidence that the mechanistic diversity of polyketide and non-ribosomal peptide assembly is considerably greater than that currently recognized [14], [64], and thus it can be expected that the NaPDoS classification system will need to evolve as new phylogenetic lineages are linked to specific biochemical functions and enzyme architectures. There is considerable preliminary evidence that the classes defined here will be further delineated once more experimentally characterized sequence data is obtained. For example, the current KS1 clade includes traditional starter KSs (KSQ) as well as domains from the salinosporamide (stro1024) and jamaicamide (JamE) pathways, which are involved in the incorporation of unusual extender units [8], [65]. Likewise, the Type II clade includes deeply branching KS domains derived from CurC and JamG that are predicted to be involved in decarboxylation as opposed to condensation reactions [65], [66]. A third example is the Iterative (a) class, which include traditional iterative KSs as well as those involved in the biosynthesis of polycyclic tetramate macrolactams [67]. Finally, the *trans*-AT (b) clade is comprised of KS sequenced derived from what appears to be an evolutionarily independent lineage of *trans*-AT sequences as well as genes associated with *beta*-branching [28], [55]. Despite the potential oversimplification of the current classification system, it provides a useful method to estimate the numbers and functional types of biosynthetic genes present in complex data sets.

Despite poor assembly, a large number and diversity of KS and C domains were detected among the four draft *Salinispora* genome sequences. Seventeen of these domains were not observed in either of the two complete *Salinispora* genomes providing evidence of the considerable biosynthetic variability that may occur among closely related strains. In addition, two C domains fell into the “Modified AA” clade, a lineage whose biochemical function has yet to be experimentally characterized. While the metagenomic datasets revealed similar total numbers of KS domains, the classification of those domains revealed dramatic differences in functional types. Analyses such as these provide insight into the potential significance of secondary chemistry in mediating population and community dynamics while at the same time identifying environments that can be prioritized for secondary metabolite discovery efforts.

Traditional natural product discovery paradigms have become increasingly inefficient [68] and are rapidly moving towards approaches that capitalize on access to DNA sequence data [69]. NaPDoS is a publically available bioinformatic tool that capitalizes on the well-established phylogenetic relationships of KS and C domains. It provides a rapid method to make informed interpretations of secondary metabolism based on small sequence tags extracted from a variety of data types including poorly assembled, next generation datasets. A major application of NaPDoS is the exploration of sequence space and the identification of new domain lineages, which have a high probability of being associated with new mechanisms of secondary metabolite biosynthesis. Prioritizing these lineages for experimental characterization will facilitate the discovery of new biochemistry and represents a rationale approach to secondary metabolite discovery.

At present, NaPDoS is optimized for the identification and classification of bacterial PKS and NRPS genes. Nonetheless, it is possible for NaPDoS to identify eukaryotic KS and C domains given their shared evolutionary history with prokaryotic homologs. The results obtained for non-bacterial sequences should be interpreted with caution however, as the reference database has not been adequately populated with these sequences to provide a robust classification system. Future plans include the expansion of NaPDoS to include additional eukaryotic sequences and subgroups within the FAS and PUFA lineages, the later of which were recently shown to cluster phylogenetically based on functional type [70]. Additional goals are to include type III PKSs, which were originally found in plants but are now known to occur in a wide range of bacteria [71]. These PKSs are distantly related to types I and II and thus will require a separate alignment and analysis pipeline. The inclusion of additional secondary metabolite families, such as terpenoids, alkaloids, and ribosomal peptides, is also conceivable.

## Supporting Information

Table S1 **NaPDoS derived KS and C domains from the** ***S. avermitilis*** **MA-4680 genome.**
(DOC)

Table S2 **NaPDoS results for six** ***Salinispora*** **genomes.**
(DOC)

Table S3 **KS and C domains detected in four draft** ***Salinispora*** **genomes.**
(DOC)

Table S4 **NaPDoS and antiSMASH-derived KS and C domains.**
(DOCX)

Table S5 **NaPDoS KS results for metagenomic data sets.**
(DOC)

Table S6 **KS domains detected in the whale fall metagenomic data set.**
(DOC)

Table S7 **KS domains detected in the Minnesota farm soil data set.**
(DOC)
