# Supplementary material for: The Natural Product Domain Seeker NaPDoS: A Phylogeny Based Bioinformatic Tool to Classify Secondary Metabolite Gene Diversity
Source: PLoS One. 2012 Mar 29;7(3):e34064. doi: 10.1371/journal.pone.0034064 (PMC3315503; doi:10.1371/journal.pone.0034064)
Supplement: Table S1 — NaPDoS derived KS and C domains from the S. avermitilis MA-4680 genome. (DOC) [file pone.0034064.s001.doc]

**Table S1.** NaPDoS derived KS and C domains from the *S. avermitilis* MA-4680 genome.

| Domain # | NaPDoS  database match | %  ID | Length | e-value | NaPDoS  pathway | Domain classification | Locus  tag^a^ | Predicted compound |
| --- | --- | --- | --- | --- | --- | --- | --- | --- |
| KS1 | AlnL_ACI88861_KSa | 43 | 326 | 2.00E-51 | alnumycin | type II | SAV_2292 | fatty acid |
| KS2 | AlnM_ACI88862_KSb | 47 | 407 | 8.00E-81 | alnumycin | type II | SAV__2839 | spore pigment |
| KS3 | CALO5_12183629_i1 | 48 | 429 | 8.00E-102 | calicheamicin | Iterative | SAV_2893 | oligomycin |
| KS4 | AlnL_ACI88861_KSa | 37 | 372 | 3.00E-43 | alnumycin | type II | SAV_2944 | fatty acid |
| KS5 | AveA1_Q9S0R8_1mod | 100 | 226 | 2.00E-127 | avermectin | Modular | SAV_943 | avermectin |
| KS6 | AveA1_Q9S0R8_2mod | 100 | 222 | 1.00E-126 | avermectin | Modular | SAV_943 | avermectin |
| KS7 | AveA2_Q9S0R7_1mod | 100 | 223 | 6.00E-122 | avermectin | Modular | SAV_943 | avermectin |
| KS8 | AveA2_Q9S0R7_2mod | 100 | 222 | 5.00E-126 | avermectin | Modular | SAV_943 | avermectin |
| KS9 | AveA2_Q9S0R7_3mod | 100 | 222 | 2.00E-126 | avermectin | Modular | SAV_943 | avermectin |
| KS10 | AveA2_Q9S0R7_4mod | 100 | 224 | 4.00E-120 | avermectin | Modular | SAV_943 | avermectin |
| KS11 | sporepig_NP824014_SP | 100 | 239 | 8.00E-141 | spore pigment | type II | SAV_2838 | spore pigment |
| KS12 | Strep_ZP_06279092_i1 | 46 | 427 | 2.00E-90 | unknown | Iterative | SAV_1249 | PK-NRP hybrid |
| KS13 | Avi_AAK83194_i1v2 | 48 | 436 | 2.00E-110 | avilamycin | Iterative | SAV_2892 | oligomycin |
| KS14 | Avi_AAK83194_i1v2 | 51 | 436 | 4.00E-113 | avilamycin | Iterative | SAV_2892 | oligomycin |
| KS15 | HSAF_ABL86391_i1V2 | 39 | 462 | 1.00E-80 | HSAF | Iterative | SAV_100 | polyketide |
| KS16 | Stro2795_1 | 54 | 211 | 5.00E-62 | ST Sid 3 | KS | SAV_3665 | polyketide |
| KS17 | Avi_AAK83194_i1v2 | 48 | 436 | 7.00E-94 | avilamycin | Iterative | SAV_2898 | oligomycin |
| KS18 | Avi_AAK83194_i1v2 | 51 | 436 | 2.00E-104 | avilamycin | Iterative | SAV_2898 | oligomycin |
| KS19 | Avi_AAK83194_i1v2 | 47 | 441 | 8.00E-102 | avilamycin | Iterative | SAV_2898 | oligomycin |
| KS20 | Avi_AAK83194_i1v2 | 52 | 436 | 7.00E-109 | avilamycin | Iterative | SAV_2864 | oligomycin |
| KS21 | Avi_AAK83194_i1v2 | 50 | 436 | 2.00E-97 | avilamycin | Iterative | SAV_2864 | oligomycin |
| KS22 | Avi_AAK83194_i1v2 | 48 | 436 | 2.00E-102 | avilamycin | Iterative | SAV_2864 | oligomycin |
| KS23 | AlnL_ACI88861_KSa | 66 | 366 | 2.00E-137 | alnumycin | type II | SAV_2376 | polyketide |
| KS24 | bleom_AAG02357_RH | 51 | 428 | 1.00E-104 | bleomycin | Hybrid | SAV_845 | NRP |
| KS25 | Avi_AAK83194_i1v2 | 47 | 436 | 1.00E-105 | avilamycin | Iterative | SAV_416 | filipin |
| KS26 | Avi_AAK83194_i1v2 | 50 | 436 | 4.00E-110 | avilamycin | Iterative | SAV_416 | filipin |
| KS27 | Avi_AAK83194_i1v2 | 49 | 436 | 3.00E-113 | avilamycin | Iterative | SAV_416 | filipin |
| KS28 | Avi_AAK83194_i1v2 | 50 | 436 | 4.00E-112 | avilamycin | Iterative | SAV_416 | filipin |
| KS29 | Avi_AAK83194_i1v2 | 48 | 436 | 3.00E-106 | avilamycin | Iterative | SAV_416 | filipin |
| KS30 | Avi_AAK83194_i1v2 | 49 | 436 | 3.00E-116 | avilamycin | Iterative | SAV_416 | filipin |
| KS31 | Stro3381_1 | 63 | 237 | 5.00E-74 | unknown | FAS | SAV_5785 | fatty acid |
| KS32 | KirAIV_CAN89634_11T | 44 | 438 | 8.00E-84 | kirromycin | trans-AT | SAV_7362 | polyketide |
| KS33 | KirAIV_CAN89634_11T | 38 | 472 | 9.00E-66 | kirromycin | trans-AT | SAV_7361 | polyketide |
| KS34 | VirF_BAF50722_5T | 38 | 208 | 3.00E-26 | virginiamycin | trans-AT | SAV_3667 | polyketide |
| KS35 | AlnL_ACI88861_KSa | 36 | 276 | 2.00E-22 | alnumycin | type II | SAV_3660 | polyketide |
| KS36 | Strep_ZP_06279092_i1 | 46 | 430 | 1.00E-102 | unknown | iterative | SAV_7184 | polyketide |
| KS37 | HSAF_ABL86391_i1V2 | 48 | 427 | 1.00E-95 | HSAF | iterative | SAV_2899 | oligomycin |
| KS38 | Avi_AAK83194_i1v2 | 51 | 436 | 2.00E-114 | avilamycin | iterative | SAV_2899 | oligomycin |
| KS39 | CALO5_12183629_i1 | 51 | 427 | 6.00E-109 | calicheamicin | Iterative | SAV_2899 | oligomycin |
| KS40 | Avi_AAK83194_i1v2 | 51 | 435 | 3.00E-113 | avilamycin | Iterative | SAV_2899 | oligomycin |
| KS41 | Avi_AAK83194_i1v2 | 51 | 436 | 4.00E-111 | avilamycin | Iterative | SAV_2899 | oligomycin |
| KS42 | Avi_AAK83194_i1v2 | 51 | 435 | 3.00E-112 | avilamycin | Iterative | SAV_2899 | oligomycin |
| KS43 | Avi_AAK83194_i1v2 | 50 | 436 | 1.00E-96 | avilamycin | Iterative | SAV_1551 | polyketide |
| KS44 | CALO5_12183629_i1 | 50 | 428 | 7.00E-113 | calicheamicin | Iterative | SAV_1551 | polyketide |
| KS45 | Avi_AAK83194_i1v2 | 50 | 438 | 5.00E-114 | avilamycin | Iterative | SAV_410 | filipin |
| KS46 | AlnL_ACI88861_KSa | 35 | 142 | 4.00E-08 | alnumycin | type II | SAV_3663 | aromatic polyketide |
| KS47 | Avi_AAK83194_i1v2 | 48 | 436 | 2.00E-102 | avilamycin | Iterative | SAV_2895 | oligomycin |
| KS48 | Avi_AAK83194_i1v2 | 47 | 447 | 3.00E-103 | avilamycin | Iterative | SAV_2895 | oligomycin |
| KS50 | AlnM_ACI88862_KSb | 53 | 405 | 3.00E-104 | alnumycin | type II | SAV_2375 | polyketide |
| KS51 | KirAI_CAN89631_1T | 46 | 425 | 6.00E-84 | kirromycin | trans-AT | SAV_2368 | polyketide |
| KS52 | Avi_AAK83194_i1v2 | 52 | 438 | 2.00E-112 | avilamycin | iterative | SAV_2368 | polyketide |
| KS53 | Avi_AAK83194_i1v2 | 50 | 436 | 5.00E-108 | avilamycin | iterative | SAV_2368 | polyketide |
| KS54 | CALO5_12183629_i1 | 39 | 426 | 3.00E-52 | calicheamicin | Iterative | SAV_2281 | polyketide |
| KS55 | AveA4_Q9S0R3_1mod | 100 | 222 | 8.00E-118 | avermectin | Modular | SAV_943 | avermectin |
| KS56 | AveA4_Q9S0R3_2mod | 100 | 222 | 6.00E-125 | avermectin | Modular | SAV_943 | avermectin |
| KS57 | AveA4_Q9S0R3_3mod | 100 | 222 | 1.00E-125 | avermectin | Modular | SAV_943 | avermectin |
| KS58 | AveA3_Q9S0R4_1mod | 100 | 222 | 7.00E-104 | avermectin | modular | SAV_943 | avermectin |
| KS59 | AveA3_Q9S0R4_2mod | 100 | 223 | 1.00E-126 | avermectin | modular | SAV_943 | avermectin |
| KS60 | AveA3_Q9S0R4_3mod | 100 | 222 | 3.00E-126 | avermectin | modular | SAV_943 | avermectin |
| KS61 | HSAF_ABL86391_i1V2 | 49 | 424 | 2.00E-111 | HSAF | iterative | SAV_419 | filipin |
| KS62 | Avi_AAK83194_i1v2 | 49 | 435 | 2.00E-106 | avilamycin | iterative | SAV_419 | filipin |
| KS63 | Avi_AAK83194_i1v2 | 48 | 436 | 2.00E-106 | avilamycin | iterative | SAV_419 | filipin |
| KS64 | Avi_AAK83194_i1v2 | 49 | 435 | 3.00E-116 | avilamycin | iterative | SAV_419 | filipin |
| KS65 | Avi_AAK83194_i1v2 | 50 | 437 | 3.00E-112 | avilamycin | iterative | SAV_419 | filipin |
| KS66 | Avi_AAK83194_i1v2 | 48 | 436 | 5.00E-113 | avilamycin | iterative | SAV_415 | filipin |
| KS67 | Avi_AAK83194_i1v2 | 48 | 437 | 2.00E-111 | avilamycin | iterative | SAV_415 | filipin |
| C1 | cyclo1_C7_LCL | 27 | 295 | 5.00E-17 | cyclosporin | LCL | SAV_859 | NRP |
| C2 | act3_C3_LCL | 39 | 192 | 1.00E-22 | actinomycin | LCL | SAV_869 | NRP |
| C3 | syrin1_C6_LCL | 32 | 300 | 5.00E-29 | syringomycin | LCL | SAV_857 | NRP |
| C4 | ituri1_C3_LCL | 27 | 245 | 2.00E-15 | iturin | LCL | SAV_1551 | polyketide |
| C5 | bacil2_C1_start | 47 | 293 | 5.00E-77 | bacillibactin | starter | SAV_603 | NRP |
| C6 | syrin1_C6_LCL | 44 | 298 | 4.00E-60 | syringomycin | LCL | SAV_3643 | NRP |
| C7 | micro1_C1 | 36 | 302 | 2.00E-51 | microcystin | Mod.AA | SAV_3197 | NRP |
| C8 | syrin1_C6_LCL | 40 | 298 | 7.00E-56 | syringomycin | LCL | SAV_3159 | NRP |
| C9 | act3_C3_LCL | 49 | 295 | 1.00E-63 | actinomycin | LCL | SAV_865 | NRP |
| C10 | syrin1_C9_LCL | 38 | 303 | 1.00E-48 | syringomycin | LCL | SAV_852 | NRP |
| C11 | micro3_C1_LCL | 28 | 220 | 3.00E-17 | microcystin | LCL | SAV_847 | NRP |
| C12 | Sare2407_1 | 33 | 295 | 2.00E-31 | pksnrps2 | LCL | SAV_3647 | NRP |
| C13 | cdaps2_C2_LCL | 47 | 306 | 5.00E-60 | Ca-dependent antibiotic | LCL | SAV_3642 | NRP |
| C14 | micro1_C1 | 35 | 293 | 1.00E-34 | microcystin | Mod.AA | SAV_3642 | NRP |
| C15 | micro1_C1 | 34 | 293 | 2.00E-36 | microcystin | Mod.AA | SAV_3642 | NRP |

a) as defined in the *S. avermitilis* MA-4680 genome sequence.
