# Supplementary material for: The Natural Product Domain Seeker NaPDoS: A Phylogeny Based Bioinformatic Tool to Classify Secondary Metabolite Gene Diversity
Source: PLoS One. 2012 Mar 29;7(3):e34064. doi: 10.1371/journal.pone.0034064 (PMC3315503; doi:10.1371/journal.pone.0034064)
Supplement: Table S2 — NaPDoS results for six Salinispora genomes. (DOC) [file pone.0034064.s002.doc]

**Table S2.** NaPDoS results for six *Salinispora* genomes.

| Species | Strain | Size (Mb) | Contigs | KS | KS class^a^ | | | | | | | C  Total | C class^b^ | | | | |
| --- | --- | --- | --- | --- | --- | --- | --- | --- | --- | --- | --- | --- | --- | --- | --- | --- | --- |
|  |  |  |  | Total | Ene | II | Cis | Iter | Hyb | KS1 | FA |  | LCL | Cyc | Starter | DCL | Mod |
| *S. arenicola* | CNS-205 | 5.1 | 1 | 33 | 2 | 4 | 20 | 3 | 1 | 1 | 3 | 24 | 20 | 3 | 0 | 0 | 1 |
| *S. tropica* | CNB-440 | 5.7 | 1 | 28 | 2 | 8 | 12 | 0 | 2 | 1 | 3 | 16 | 8 | 7 | 1 | 0 | 0 |
| *S. arenicola* | CNT-088 | 5.4 | 2304 | 32 | 2 | 1 | 21 | 4 | 2 | 1 | 1 | 16 | 13 | 2 | 0 | 0 | 1 |
| *S. arenicola* | CNH-643 | 4.8 | 3823 | 29 | 1 | 1 | 21 | 1 | 2 | 1 | 2 | 9 | 6 | 1 | 0 | 1 | 1 |
| *“S. pacifica”* | CNT-133 | 4.5 | 5214 | 32 | 1 | 4 | 19 | 1 | 1 | 2 | 4 | 6 | 6 | 0 | 0 | 0 | 0 |
| *“S. pacifica”* | CNS-143 | 4.1 | 5260 | 25 | 1 | 1 | 18 | 0 | 3 | 2 | 0 | 7 | 3 | 2 | 1 | 1 | 0 |

a) Ene = enediyne, II = type II, cis = *cis*-AT modular, Iter = iterative, Hyb = hybrid, FA = fatty acid.

b) Cyc = cyclization, Mod = “modified amino acid”.
