# Supplementary material for: The Natural Product Domain Seeker NaPDoS: A Phylogeny Based Bioinformatic Tool to Classify Secondary Metabolite Gene Diversity
Source: PLoS One. 2012 Mar 29;7(3):e34064. doi: 10.1371/journal.pone.0034064 (PMC3315503; doi:10.1371/journal.pone.0034064)
Supplement: Table S3 — KS and C domains detected in four draft Salinispora genomes. (DOC) [file pone.0034064.s003.doc]

**Table S3.**  KS and C domains detected in four draft *Salinispora* genomes.

| Pathway  name^a^ | Domain classification | Predicted  compound^b^ | *S. arenicola*  CNS-205 | *S. tropica*  CNB-440 | *S. arenicola*  CNT-088 | *S. arenicola*  CNH-643 | *S. pacifica*  CNT-133 | *S. pacifica*  CNS-143 |
| --- | --- | --- | --- | --- | --- | --- | --- | --- |
| PKS1A | enediyne | 9 membered enediyne | X | - | X | X | - | - |
| PKS2 | type II | polyketide | X | - | - | - | - | - |
| Rif | modular | rifamycin and saliniketals | X | - | X | X | - | - |
| PKS3A | iterative | calicheamicin-related fragment A | X | - | X | X | - | - |
| Sid1 | hybrid | yersiniabactin related siderophore | X | X | X | X | - | - |
| PKS3B | enediyne | calicheamicin-related fragment B | X | - | X | - | - | - |
| PKS4 | type II | aromatic polyketide | X | X | X | X | X | - |
| PKSNRPS2 | modular | ND SApksnrps2 | X | - | X | - | - | - |
| PKS5 | modular | macrolide | X | - | X | X | - | - |
| lym | modular | **lymphostin** | X | X | X | X | - | X |
| pks1C | iterative | kedarcin related fragment C | X | - | X | - | - | - |
| STpks1 | enediyne | 10 membered enediyne STpks1 | - | X | - | - | - | - |
| sal | KS1, hybrid | salinisporamide | - | X | - | - | X | - |
| STPKS2 | type II | glycosylated decaketide | - | X | - | - | X | 1 |
| spo | enediyne | **sporolide** | - | X | - | - | X | - |
| slm | modular | **salinilactam** | - | X | - | - | X | X |
| cya | enediyne | **cyanosporaside** | - | - | - | - | - | X |
| STSid3 | type II | dihydroaeruginoic acid related siderophore | - | X | - | - | - | - |
| tyl | modular | **tylactone** | - | - | - | - | X | - |
| fa | fatty acid | fatty acid | X | X | X | X | X | - |
| PKS7 | modular | polyketide | - | - | - | X | X | X |
| PKS8 | hybrid | NRP/PK hybrid | - | - | - | X | - | - |
| PKS9 | modular | polyketide | - | - | - | - | - | X |
| PKS10 | fatty acid | fatty acid | - | - | - | - | X | - |
| PKS11 | iterative | polyketide | - | - | - | - | X | - |
| PKS12 | modular | polyketide | - | - | - | - | X | - |
| PKS13 | KS1 | polyketide | - | - | - | - | - | - |
| PKS14 | KS1 | polyketide | - | - | - | - | - | X |
| PKS15 | hybrid | NRP/PK hybrid | - | - | - | - | - | X |
| PKS16 | modular | polyketide | - | - | - | - | - | X |
| PKS17 | fatty acid | fatty acid | - | - | - | X | X | - |
| PKS18 | hybrid | NRP/PK hybrid | - | - | - | - | - | X |
| PKS19 | modular | FD-891-like | - | - | - | - | - | X |
| PKS20 | modular | polyketide | - | - | - | - | - | X |
| PKS21 | hybrid | NRP/PK hybrid | - | - | - | - | - | X |
| NRPS 1 | LCL, modified AA | pentapeptide | X | - | X | X | - | - |
| Sid1 | cyclization | yersiniabactin-related | X | X | X | X | - | - |
| PKS1B | LCL | kedarcidin-related | X | - | X | X | - | - |
| PKSNRPS2 | LCL | polyketide/non-ribosomal peptide | X | - | X | - | - | - |
| NRPS2 | LCL | tetrapeptide | X | - | X | - | - | - |
| NRPS3 | LCL | dipeptide | - | X | - | - | - | - |
| Cym | LCL | **cyclomarin** | X | - | - | - | - | - |
| NRPS4 | LCL | tetrapeptide | X | X | X | - | X | X |
| Sal | LCL | **salinosporamide** | - | X | - | - | X | - |
| Sid3 | LCL | dihydroaeruginoic-acid related | - | X | - | - | - | - |
| Sid4 | cyclization, LCL | coelibactin-related siderophore | - | X | - | - | - | - |
| Spo | LCL | **sporolide** | - | X | - | - | - | - |
| NRPS5 | LCL | NRP | - | - | X | - | - | - |
| NRPS6 | LCL | NRP | - | - | X | - | - | - |
| NRPS7 | LCL | NRP | - | - | X | - | - | - |
| NRPS8 | DCL | NRP | - | - | - | - | - | X |
| NRPS9 | LCL | NRP | - | - | - | - | X | - |
| NRPS10 | LCL | NRP | - | - | - | - | X | - |
| NRPS11 | LCL | NRP | - | - | - | - | X | - |
| NRPS12 | cyclization | NRP | - | - | - | - | - | X |
| NRPS13 | cyclization | NRP | - | - | - | - | - | X |
| NRPS14 | LCL | NRP | - | - | - | X | - | - |
| NRPS15 | starter | NRP | - | - | - | - | - | X |
| NRPS16 | DCL | NRP | - | - | - | X | - | - |

a) Pathway names and associated compounds are as previously reported (Penn et al., 2009). In cases of <90% sequence identity to an experimentally characterized pathway, domains were given PKS and NRPS numbers.

b) Compounds in bold have been isolated from at least one of the strains.
