# Supplementary material for: The Natural Product Domain Seeker NaPDoS: A Phylogeny Based Bioinformatic Tool to Classify Secondary Metabolite Gene Diversity
Source: PLoS One. 2012 Mar 29;7(3):e34064. doi: 10.1371/journal.pone.0034064 (PMC3315503; doi:10.1371/journal.pone.0034064)
Supplement: Table S4 — NaPDoS and antiSMASH-derived KS and C domains. (DOCX) [file pone.0034064.s004.docx]

**Table S4.** NaPDoS and antiSMASH-derived KS and C domains.

|  |  | KS domains | |  | C domains | |
| --- | --- | --- | --- | --- | --- | --- |
| Species | Strain | antiSMASH | NaPDoS^a^ |  | antiSMASH | NaPDoS^b^ |
| *S. arenicola* | CNH-643 | 27 | 34 |  | 16 | 15 |
| *S. arenicola* | CNT-088 | 25 | 30 |  | 13 | 14 |
| *S. pacifica* | CNS-143 | 10 | 16 |  | 10 | 9 |
| *S. pacifica* | CNT-133 | 7 | 17 |  | 7 | 8 |

^a^KS domains associated with fatty acid biosynthesis were manually removed from the NaPDoS totals as antiSMASH does this automatically.

^b^The NaPDoS C domain cut-off was set to 100 amino acids to be more comparable with antiSMASH.
