# Supplementary material for: The Natural Product Domain Seeker NaPDoS: A Phylogeny Based Bioinformatic Tool to Classify Secondary Metabolite Gene Diversity
Source: PLoS One. 2012 Mar 29;7(3):e34064. doi: 10.1371/journal.pone.0034064 (PMC3315503; doi:10.1371/journal.pone.0034064)
Supplement: Table S5 — NaPDoS KS results for metagenomic data sets. (DOC) [file pone.0034064.s005.doc]

**Table S5.** NaPDoS KS results for metagenomic data sets.

|  |  |  |  |  |  |  | Class |  |  |  |  |
| --- | --- | --- | --- | --- | --- | --- | --- | --- | --- | --- | --- |
| Dataset | Total  KS domains | Distinct  KS domains | Fatty  acid | Type II | Hybrid | Modular | *Trans*-  AT | KS1 | Iterative | PUFA | Non-  KS |
| Whale fall | 129 | 42 | 27 | 1 | 1 | 0 | 1 | 0 | 0 | 1 | 11 |
| Farm soil | 128 | 127 | 43 | 15 | 11 | 20 | 8 | 4 | 4 | 0 | 22 |
