# Supplementary material for: The Natural Product Domain Seeker NaPDoS: A Phylogeny Based Bioinformatic Tool to Classify Secondary Metabolite Gene Diversity
Source: PLoS One. 2012 Mar 29;7(3):e34064. doi: 10.1371/journal.pone.0034064 (PMC3315503; doi:10.1371/journal.pone.0034064)
Supplement: Table S6 — KS domains detected in the whale fall metagenomic data set. (DOC) [file pone.0034064.s006.doc]

**Table S6.** KS domains detected in the whale fall metagenomic data set.

| Query KS | | NaPDoS database match | | | | | |
| --- | --- | --- | --- | --- | --- | --- | --- |
| KS | Domain class | Database name | Percent identity | Align length | e-value | Pathway product | Domain class |
| 1 | non KS | PfaA_Shewanella_PUFA | 44 | 203 | 2.00E-52 | polyunsaturated fatty acid | PUFA |
| 2 | FAS | FabF_Bacillus_FAS | 47 | 309 | 6.00E-69 | fatty acid synthesis | FAS |
| 3 | FAS | FabB_Ecoli_FAS | 72 | 240 | 9.00E-98 | fatty acid synthesis | FAS |
| 4 | FAS | LnmJ_AF484556_4T | 41 | 94 | 2.00E-14 | leinamycin | trans |
| 5 | FAS | FabB_Ecoli_FAS | 42 | 280 | 2.00E-53 | fatty acid synthesis | FAS |
| 6 | non KS | PfaA_Shewanella_PUFA | 88 | 255 | 8.00E-139 | polyunsaturated fatty acid | PUFA |
| 7 | PUFA | PfaC_Shewanella_PUFA | 36 | 140 | 9.00E-24 | polyunsaturated fatty acid | PUFA |
| 8 | FAS | FabF_Ecoli_FAS | 38 | 267 | 3.00E-22 | fatty acid synthesis | FAS |
| 9 | type II | VicB_BAD08358_1KSB | 36 | 108 | 5.00E-08 | vicenistatin | modular |
| 10 | non KS | FabF_Bacillus_FAS | 33 | 320 | 1.00E-35 | fatty acid synthesis | FAS |
| 11 | FAS | FabF_Bacillus_FAS | 53 | 254 | 5.00E-71 | fatty acid synthesis | FAS |
| 12 | non KS | FabF_Bacillus_FAS | 37 | 131 | 9.00E-16 | fatty acid synthesis | FAS |
| 13 | non KS | FabF_Bacillus_FAS | 50 | 236 | 9.00E-60 | fatty acid synthesis | FAS |
| 14 | FAS | FabF_Bacillus_FAS | 58 | 210 | 1.00E-54 | fatty acid synthesis | FAS |
| 15 | non KS | FabF_Bacillus_FAS | 51 | 230 | 4.00E-56 | fatty acid synthesis | FAS |
| 16 | hybrid | bleom_AAG02357_H | 54 | 197 | 2.00E-57 | bleomycin | hybrid |
| 17 | FAS | FabF_Bacillus_FAS | 28 | 104 | 2.00E-07 | fatty acid synthesis | FAS |
| 18 | FAS | FabB_Ecoli_FAS | 77 | 193 | 4.00E-85 | fatty acid synthesis | FAS |
| 19 | non KS | Nostoc_glycolipid_PUFA | 39 | 136 | 6.00E-21 | heterocyst glycolipid | PUFA |
| 20 | FAS | FabF_Bacillus_FAS | 55 | 134 | 1.00E-39 | fatty acid synthesis | FAS |
| 21 | FAS | FabB_Ecoli_FAS | 64 | 214 | 1.00E-71 | fatty acid synthesis | FAS |
| 22 | FAS | FabB_Ecoli_FAS | 36 | 143 | 1.00E-14 | fatty acid synthesis | FAS |
| 23 | non KS | bleom_AAG02357_H | 65 | 104 | 3.00E-39 | bleomycin | hybrid |
| 24 | FAS | FabF_Bacillus_FAS | 41 | 248 | 2.00E-44 | fatty acid synthesis | FAS |
| 25 | FAS | FabF_Bacillus_FAS | 37 | 155 | 9.00E-30 | fatty acid synthesis | FAS |
| 26 | FAS | FabF_Bacillus_FAS | 34 | 190 | 3.00E-23 | fatty acid synthesis | FAS |
| 27 | FAS | FabF_Bacillus_FAS | 56 | 214 | 1.00E-52 | fatty acid synthesis | FAS |
| 28 | FAS | KirAII_CAN89632_4T | 36 | 121 | 9.00E-10 | kirromycin | trans |
| 29 | FAS | FabB_Ecoli_FAS | 72 | 193 | 1.00E-75 | fatty acid synthesis | FAS |
| 30 | non KS | FabF_Bacillus_FAS | 43 | 154 | 1.00E-25 | fatty acid synthesis | FAS |
| 31 | FAS | FabF_Bacillus_FAS | 54 | 255 | 3.00E-63 | fatty acid synthesis | FAS |
| 32 | FAS | FabF_Bacillus_FAS | 49 | 185 | 2.00E-43 | fatty acid synthesis | FAS |
| 33 | FAS | FabF_Bacillus_FAS | 57 | 244 | 4.00E-79 | fatty acid synthesis | FAS |
| 34 | FAS | FabF_Bacillus_FAS | 50 | 125 | 2.00E-31 | fatty acid synthesis | FAS |
| 35 | FAS | FabB_Ecoli_FAS | 29 | 210 | 4.00E-16 | fatty acid synthesis | FAS |
| 36 | non KS | FabF_Bacillus_FAS | 44 | 186 | 1.00E-37 | fatty acid synthesis | FAS |
| 37 | non KS | FabF_Ecoli_FAS | 46 | 212 | 2.00E-44 | fatty acid synthesis | FAS |
| 38 | FAS | FabF_Bacillus_FAS | 33 | 244 | 3.00E-20 | fatty acid synthesis | FAS |
| 39 | FAS | FabF_Bacillus_FAS | 52 | 157 | 3.00E-46 | fatty acid synthesis | FAS |
| 40 | FAS | FabF_Bacillus_FAS | 51 | 262 | 1.00E-65 | fatty acid synthesis | FAS |
| 41 | FAS | FabF_Bacillus_FAS | 57 | 210 | 3.00E-65 | fatty acid synthesis | FAS |
| 42 | trans | mycos_Q9R9J1_T | 43 | 287 | 1.00E-59 | mycosubtilin | trans |
|  |  |  |  |  |  |  |  |
