# Supplementary material for: The Natural Product Domain Seeker NaPDoS: A Phylogeny Based Bioinformatic Tool to Classify Secondary Metabolite Gene Diversity
Source: PLoS One. 2012 Mar 29;7(3):e34064. doi: 10.1371/journal.pone.0034064 (PMC3315503; doi:10.1371/journal.pone.0034064)
Supplement: Table S7 — KS domains detected in the Minnesota farm soil data set. (DOC) [file pone.0034064.s007.doc]

**Table S7.** KS domains detected in the Minnesota farm soil data set.

| Query KS | | NaPDoS database match | | | | | |
| --- | --- | --- | --- | --- | --- | --- | --- |
| KS | Domain  class | Database name | Percent  identity | Align  length | e-value | Pathway product | Domain class |
| 1 | FAS | Strep_ZP_06279092_i | 54 | 87 | 6.00E-22 | unknown | iterative |
| 2 | FAS | FabF_Bacillus_FAS | 55 | 288 | 8.00E-95 | fatty acid synthesis | FAS |
| 3 | modular | Avi_AAK83194_i | 55 | 254 | 3.00E-73 | avilamycin | iterative |
| 4 | FAS | FabF_Bacillus_FAS | 58 | 280 | 1.00E-97 | fatty acid synthesis | FAS |
| 5 | non KS | MxaC_Q93TW9_3KSB | 37 | 63 | 2.00E-06 | myxalamid | modular |
| 6 | typeII | FabF_Bacillus_FAS | 35 | 364 | 8.00E-42 | fatty acid synthesis | FAS |
| 7 | iterative | CALO5_12183629_i | 55 | 209 | 1.00E-45 | calicheamicin | iterative |
| 8 | hybrid | bleom_AAG02357_H | 55 | 221 | 5.00E-67 | bleomycin | hybrid |
| 9 | non KS | FabF_Bacillus_FAS | 49 | 179 | 5.00E-40 | fatty acid synthesis | FAS |
| 10 | modular | Avi_AAK83194_i | 52 | 206 | 6.00E-58 | avilamycin | iterative |
| 11 | typeII | FabF_Bacillus_FAS | 33 | 304 | 6.00E-37 | fatty acid synthesis | FAS |
| 12 | non KS | yersi_YP_070123_H | 36 | 100 | 2.00E-15 | yersiniabactin | hybrid |
| 13 | KS1 | HSAF_ABL86391_i | 53 | 258 | 7.00E-73 | HSAF | iterative |
| 14 | typeII | FabF_Bacillus_FAS | 43 | 210 | 9.00E-44 | fatty acid synthesis | FAS |
| 15 | FAS | FabF_Bacillus_FAS | 54 | 257 | 2.00E-66 | fatty acid synthesis | FAS |
| 16 | trans | LnmJ_AF484556_2T | 52 | 296 | 1.00E-85 | leinamycin | trans |
| 17 | typeII | FabF_Bacillus_FAS | 54 | 234 | 9.00E-74 | fatty acid synthesis | FAS |
| 18 | FAS | FabF_Bacillus_FAS | 54 | 240 | 8.00E-76 | fatty acid synthesis | FAS |
| 19 | trans | LnmJ_AF484556_2T | 50 | 346 | 3.00E-94 | leinamycin | trans |
| 20 | FAS | FabF_Streptomyces_FAS | 34 | 273 | 2.00E-31 | fatty acid synthesis | FAS |
| 21 | trans | LnmJ_AF484556_1T | 61 | 283 | 5.00E-88 | leinamycin | trans |
| 22 | typeII | FabF_Bacillus_FAS | 38 | 276 | 7.00E-48 | fatty acid synthesis | FAS |
| 23 | typeII | FabF_Bacillus_FAS | 27 | 327 | 3.00E-22 | fatty acid synthesis | FAS |
| 24 | FAS | FabF_Ecoli_FAS | 55 | 362 | 4.00E-101 | fatty acid synthesis | FAS |
| 25 | non KS | LnmI_AF484556_2T | 45 | 110 | 1.00E-26 | leinamycin | trans |
| 26 | FAS | FabF_Bacillus_FAS | 43 | 243 | 2.00E-41 | fatty acid synthesis | FAS |
| 27 | modular | CALO5_12183629_i | 58 | 144 | 2.00E-45 | calicheamicin | iterative |
| 28 | non KS | KirAI_CAN89631_2T | 47 | 73 | 2.00E-13 | kirromycin | trans |
| 29 | FAS | AknB_AF257324_KSa | 40 | 330 | 1.00E-50 | aclacinomycin | typeII |
| 30 | non KS | Avi_AAK83194_i | 48 | 153 | 2.00E-34 | avilamycin | iterative |
| 31 | non KS | FabF_Ecoli_FAS | 53 | 110 | 4.00E-27 | fatty acid synthesis | FAS |
| 32 | FAS | FabF_Bacillus_FAS | 36 | 303 | 9.00E-52 | fatty acid synthesis | FAS |
| 33 | modular | HSAF_ABL86391_i | 50 | 321 | 1.00E-87 | HSAF | iterative |
| 34 | FAS | FabF_Bacillus_FAS | 59 | 137 | 6.00E-48 | fatty acid synthesis | FAS |
| 35 | modular | HSAF_ABL86391_i | 51 | 245 | 4.00E-60 | HSAF | iterative |
| 36 | FAS | FabF_Ecoli_FAS | 55 | 268 | 1.00E-87 | fatty acid synthesis | FAS |
| 37 | KS1 | HSAF_ABL86391_i | 50 | 306 | 2.00E-84 | HSAF | iterative |
| 38 | iterative | Strep_ZP_06279092_i | 55 | 219 | 2.00E-64 | unknown | iterative |
| 39 | typeII | FabF_Bacillus_FAS | 44 | 323 | 9.00E-64 | fatty acid synthesis | FAS |
| 40 | FAS | FabF_Bacillus_FAS | 60 | 201 | 2.00E-72 | fatty acid synthesis | FAS |
| 41 | typeII | FabF_Streptomyces_FAS | 39 | 143 | 4.00E-20 | fatty acid synthesis | FAS |
| 42 | modular | JamK_AAS98782_mod | 62 | 227 | 8.00E-83 | jamaicamide | modular |
| 43 | non KS | LnmJ_AF484556_3T | 48 | 86 | 9.00E-13 | leinamycin | trans |
| 44 | trans | VirA_BAF50727_4T | 45 | 317 | 5.00E-60 | virginiamycin | trans |
| 45 | FAS | FabF_Bacillus_FAS | 36 | 242 | 8.00E-35 | fatty acid synthesis | FAS |
| 46 | typeII | FabF_Bacillus_FAS | 36 | 221 | 3.00E-28 | fatty acid synthesis | FAS |
| 47 | typeII | FabF_Bacillus_FAS | 47 | 183 | 4.00E-41 | fatty acid synthesis | FAS |
| 48 | non KS | FabF_Ecoli_FAS | 48 | 173 | 3.00E-41 | fatty acid synthesis | FAS |
| 49 | modular | HSAF_ABL86391_i | 49 | 205 | 1.00E-50 | HSAF | iterative |
| 50 | modular | StiH_Q8RJX9_1KSB | 56 | 151 | 9.00E-37 | stigmatellin | modular |
| 51 | non KS | bleom_AAG02357_H | 33 | 166 | 2.00E-18 | bleomycin | hybrid |
| 52 | FAS | FabB_Ecoli_FAS | 64 | 284 | 5.00E-105 | fatty acid synthesis | FAS |
| 53 | modular | CALO5_12183629_i | 49 | 336 | 3.00E-82 | calicheamicin | iterative |
| 54 | KS1 | KirAII_CAN89632_5T | 52 | 190 | 1.00E-52 | kirromycin | trans |
| 55 | non KS | bleom_AAG02357_H | 40 | 126 | 2.00E-27 | bleomycin | hybrid |
| 56 | FAS | FabF_Bacillus_FAS | 37 | 260 | 4.00E-43 | fatty acid synthesis | FAS |
| 57 | modular | HSAF_ABL86391_i | 51 | 290 | 2.00E-83 | HSAF | iterative |
| 58 | FAS | FabF_Bacillus_FAS | 46 | 181 | 5.00E-43 | fatty acid synthesis | FAS |
| 59 | hybrid | bleom_AAG02357_H | 63 | 283 | 2.00E-92 | bleomycin | hybrid |
| 60 | FAS | FabF_Bacillus_FAS | 57 | 144 | 1.00E-45 | fatty acid synthesis | FAS |
| 61 | hybrid | bleom_AAG02357_H | 54 | 293 | 9.00E-76 | bleomycin | hybrid |
| 62 | modular | Strep_ZP_06279092_i | 48 | 244 | 5.00E-64 | unknown | iterative |
| 63 | FAS | FabF_Bacillus_FAS | 67 | 243 | 3.00E-89 | fatty acid synthesis | FAS |
| 64 | iterative | CALO5_12183629_i | 53 | 276 | 3.00E-57 | calicheamicin | iterative |
| 65 | modular | KirAIV_CAN89634_10T | 49 | 134 | 8.00E-28 | kirromycin | trans |
| 66 | KS1 | HSAF_ABL86391_i | 55 | 202 | 4.00E-58 | HSAF | iterative |
| 67 | FAS | FabF_Bacillus_FAS | 54 | 255 | 4.00E-73 | fatty acid synthesis | FAS |
| 68 | hybrid | bleom_AAG02357_H | 55 | 356 | 7.00E-105 | bleomycin | hybrid |
| 69 | FAS | FabF_Ecoli_FAS | 44 | 162 | 1.00E-33 | fatty acid synthesis | FAS |
| 70 | FAS | FabF_Bacillus_FAS | 59 | 228 | 2.00E-61 | fatty acid synthesis | FAS |
| 71 | trans | VirA_BAF50727_4T | 50 | 230 | 7.00E-59 | virginiamycin | trans |
| 72 | modular | CALO5_12183629_i | 55 | 287 | 5.00E-77 | calicheamicin | iterative |
| 73 | trans | KirAIV_CAN89634_7T | 49 | 259 | 8.00E-66 | kirromycin | trans |
| 74 | modular | COMPA_BAC20564_i | 41 | 252 | 1.00E-59 | compactin | iterative |
| 75 | hybrid | yersi_YP_070123_H | 57 | 92 | 1.00E-25 | yersiniabactin | hybrid |
| 76 | trans | LnmJ_AF484556_4T | 58 | 259 | 7.00E-88 | leinamycin | trans |
| 77 | FAS | FabF_Ecoli_FAS | 54 | 200 | 5.00E-50 | fatty acid synthesis | FAS |
| 78 | FAS | KirAIV_CAN89634_11T | 41 | 150 | 6.00E-29 | kirromycin | trans |
| 79 | non KS | Nostoc_glycolipid_PUFA | 50 | 105 | 2.00E-24 | heterocyst glycolipid | PUFA |
| 80 | modular | CALO5_12183629_i | 50 | 309 | 2.00E-80 | calicheamicin | iterative |
| 81 | FAS | FabF_Ecoli_FAS | 76 | 248 | 3.00E-100 | fatty acid synthesis | FAS |
| 82 | typeII | FabF_Bacillus_FAS | 38 | 299 | 3.00E-34 | fatty acid synthesis | FAS |
| 83 | typeII | FabF_Bacillus_FAS | 35 | 252 | 1.00E-33 | fatty acid synthesis | FAS |
| 84 | hybrid | yersi_YP_070123_H | 51 | 348 | 4.00E-103 | yersiniabactin | hybrid |
| 85 | modular | Strep_ZP_06279092_i | 50 | 218 | 1.00E-57 | unknown | iterative |
| 86 | typeII | FabF_Bacillus_FAS | 38 | 211 | 6.00E-35 | fatty acid synthesis | FAS |
| 87 | FAS | FabF_Bacillus_FAS | 54 | 245 | 3.00E-72 | fatty acid synthesis | FAS |
| 88 | non KS | FabF_Bacillus_FAS | 48 | 169 | 3.00E-36 | fatty acid synthesis | FAS |
| 89 | FAS | FabF_Bacillus_FAS | 50 | 240 | 4.00E-62 | fatty acid synthesis | FAS |
| 90 | hybrid | bleom_AAG02357_H | 61 | 266 | 2.00E-83 | bleomycin | hybrid |
| 91 | non KS | HSAF_ABL86391_i | 49 | 80 | 1.00E-20 | HSAF | iterative |
| 92 | hybrid | bleom_AAG02357_H | 61 | 287 | 2.00E-90 | bleomycin | hybrid |
| 93 | FAS | FabF_Bacillus_FAS | 33 | 152 | 1.00E-19 | fatty acid synthesis | FAS |
| 94 | FAS | FabB_Ecoli_FAS | 63 | 259 | 5.00E-88 | fatty acid synthesis | FAS |
| 95 | hybrid | bleom_AAG02357_H | 60 | 287 | 2.00E-89 | bleomycin | hybrid |
| 96 | iterative | CALO5_12183629_i | 60 | 213 | 4.00E-68 | calicheamicin | iterative |
| 97 | typeII | FabF_Bacillus_FAS | 39 | 270 | 1.00E-44 | fatty acid synthesis | FAS |
| 98 | non KS | pfaA_omega3_PUFA | 65 | 134 | 5.00E-44 | omega3_FA | PUFA |
| 99 | FAS | FabF_Bacillus_FAS | 68 | 130 | 1.00E-52 | fatty acid synthesis | FAS |
| 100 | modular | bleom_AAG02357_H | 59 | 59 | 1.00E-19 | bleomycin | hybrid |
| 101 | non KS | FabF_Bacillus_FAS | 36 | 170 | 3.00E-25 | fatty acid synthesis | FAS |
| 102 | FAS | FabF_Bacillus_FAS | 56 | 178 | 2.00E-53 | fatty acid synthesis | FAS |
| 103 | hybrid | bleom_AAG02357_H | 54 | 299 | 6.00E-90 | bleomycin | hybrid |
| 104 | modular | CALO5_12183629_i | 49 | 265 | 2.00E-65 | calicheamicin | iterative |
| 105 | non KS | MerB_ABJ97438_2KSB | 42 | 76 | 3.00E-07 | meridamycin | modular |
| 106 | FAS | bleom_AAG02357_H | 66 | 79 | 2.00E-25 | bleomycin | hybrid |
| 107 | FAS | FabF_Bacillus_FAS | 54 | 267 | 7.00E-75 | fatty acid synthesis | FAS |
| 108 | non KS | FabF_Bacillus_FAS | 48 | 125 | 5.00E-31 | fatty acid synthesis | FAS |
| 109 | FAS | FabF_Bacillus_FAS | 52 | 231 | 8.00E-66 | fatty acid synthesis | FAS |
| 110 | typeII | FabF_Bacillus_FAS | 46 | 195 | 3.00E-38 | fatty acid synthesis | FAS |
| 111 | FAS | FabF_Bacillus_FAS | 47 | 220 | 1.00E-48 | fatty acid synthesis | FAS |
| 112 | FAS | FabF_Bacillus_FAS | 59 | 257 | 2.00E-88 | fatty acid synthesis | FAS |
| 113 | FAS | FabF_Bacillus_FAS | 50 | 296 | 1.00E-76 | fatty acid synthesis | FAS |
| 114 | FAS | FabF_Ecoli_FAS | 45 | 252 | 3.00E-48 | fatty acid synthesis | FAS |
| 115 | FAS | FabF_Ecoli_FAS | 60 | 270 | 7.00E-92 | fatty acid synthesis | FAS |
| 116 | FAS | FabF_Bacillus_FAS | 62 | 191 | 4.00E-44 | fatty acid synthesis | FAS |
| 117 | non KS | HSAF_ABL86391_i | 43 | 81 | 8.00E-16 | HSAF | iterative |
| 118 | trans | KirAII_CAN89632_5T | 34 | 157 | 5.00E-14 | kirromycin | trans |
| 119 | FAS | FabF_Bacillus_FAS | 40 | 265 | 6.00E-49 | fatty acid synthesis | FAS |
| 120 | non KS | FabF_Bacillus_FAS | 38 | 210 | 1.00E-36 | fatty acid synthesis | FAS |
| 121 | FAS | FabF_Bacillus_FAS | 46 | 162 | 6.00E-41 | fatty acid synthesis | FAS |
| 122 | FAS | FabF_Bacillus_FAS | 51 | 250 | 5.00E-65 | fatty acid synthesis | FAS |
| 123 | hybrid | bleom_AAG02357_H | 58 | 262 | 3.00E-81 | bleomycin | hybrid |
| 124 | modular | KirAII_CAN89632_5T | 51 | 223 | 1.00E-61 | kirromycin | trans |
| 125 | modular | HSAF_ABL86391_i | 51 | 264 | 2.00E-75 | HSAF | iterative |
| 126 | non KS | FabF_Bacillus_FAS | 53 | 211 | 7.00E-50 | fatty acid synthesis | FAS |
| 127 | non KS | VirA_BAF50727_4T | 46 | 81 | 3.00E-17 | virginiamycin | trans |
